# Editorial: Genomics-Enabled Crop Genetics

**DOI:** 10.3389/fgene.2021.687160

**Published:** 2021-05-07

**Authors:** Yin Li, Wenqin Wang, Chuang Ma, Ray Ming

**Affiliations:** ^1^The Genetic Engineering International Cooperation Base of Chinese Ministry of Science and Technology, The Key Laboratory of Molecular Biophysics of Chinese Ministry of Education, College of Life Science and Technology, Huazhong University of Science and Technology, Wuhan, China; ^2^College of Life Sciences, Shanghai Normal University, Shanghai, China; ^3^State Key Laboratory of Crop Stress Biology for Arid Areas, Center of Bioinformatics, College of Life Sciences, Northwest A&F University, Xianyang, China; ^4^Key Laboratory of Biology and Genetics Improvement of Maize in Arid Area of Northwest Region, Ministry of Agriculture, Northwest A&F University, Xianyang, China; ^5^Department of Plant Biology, University of Illinois at Urbana-Champaign, Urbana, IL, United States

**Keywords:** crops, genomics, transcriptomics, population genetics, germplasm characterization, gene functional studies

## Introduction

In the genomics era, omics-based technologies have unprecedentedly promoted progress in plant biology, from plant growth and development, plant physiology to molecular genetic studies, and system and synthetic biology. While proteomics and metabolomics are becoming prevalent, genomics and transcriptomics are the most popular and widely used platforms for crop studies due to their rapidly decreased costs, improved sequencing quality, a broad spectrum of applications, and well-established bioinformatic tools. Genetic and functional genomic studies in crops, especially those in non-model crops, have been lagged far behind compared to those in model plant species for a couple of reasons. First, some crops can have a large, complex and polyploidy genome, such as wheat (*Triticum aestivum*) (International Wheat Genome Sequencing Consortium, 2018). Second, while a group of closely related crop species is often comparatively studied or used in breeding programs, they could have distinct genomes and/or ploidy levels, representing further technical challenges for molecular studies. For example, the peanuts include the cultivated peanut (*Arachis hypogaes*, AABB genome), the wild tetraploid peanut (*Arachis monticola*, AABB genome) and two wild diploid peanuts, *Arachis duranensis* (AA genome) and *Arachis ipaensis* (BB genome) (Bertioli et al., 2016, 2019; Chen et al., 2016, 2019; Lu et al., 2018; Yin et al., 2018, 2019; Zhuang et al., 2019). Another example is the cultivated bananas, which are interspecific or intraspecific hybrids between wild diploid *Musa acuminata* (AA genome) and *Musa balbisiana* (BB genome). They have various genotypes, including diploid (AA, BB, and AB), triploid (AAA, AAB, and ABB) and tetraploid (AAAB, AABB, ABBB) variants (D'Hont et al., 2012; Davey et al., 2013; Martin et al., 2016; Wang et al., 2019). Third, for many crops the genomic resources supporting functional studies and molecular breeding are not often available, including high-quality reference genome assemblies, high-density genetic maps, and genomics-characterized populations. Finally, in some crops (such as sorghum), genetic transformation is still challenging, and mutant resources are not well-established.

When synergistically integrated with other omics approaches, genomic technologies can be compelling for crop genetics, representing a technological basis to help mitigate or circumvent the challenges mentioned above in crop studies. The papers included in this Research Topic, Genomics-Enabled Crop Genetics, illustrate this concept. The various studies collected in this Research Topic can be summarized into three major aspects: (1) the theme of “**Genomic technologies promote germplasm characterization**” includes contributions regarding molecular identification, characterization of crop species and accessions with genomics-based methods. (2) The subject of “**Genomic technologies enhance crop population genetics**” showcases the examples of population genetic studies facilitated by the genomic approaches. (3) The topic of “**Genomic technologies enable functional mining of genomic components in crops**,” on the other hand, presents the applications in multiple genomic and transcriptomic databases. These resources are comprehensively integrated to generate functional insights into the genomic components, e.g., genes, miRNAs and *cis*-regulatory elements.

This Research Topic includes thirteen original research articles, one hypothesis and theory paper, one opinion paper and one review article, covering the following three aspects.

## Genomic Technologies Promote Germplasm Characterization

Markers of simple sequence repeats (SSR) or chloroplast DNA are often used to study the phylogenetic relationship between accessions or species within a crop genus. Taking the advantages of RNA-seq that provides sequence information about functional genes in a cost-effective and high-throughput way, Karcı et al. performed transcriptome sequencing of pistachio (*Pistacia vera*), developed 233 genic SSR markers (gSSR) and studied the phylogenetic relationship using 55 gSSR markers from nine *Pistacia* species. This study exemplifies RNA-seq as a tool to contribute to the taxonomy of crop species and their relatives. Qiu et al. assembled the five fescue taxa's chloroplast genomes, including three subspecies of *Festuca rubra*, one *Festuca brevipila*, and one *Festuca ovina*, providing resources to screen fescue germplasm accessions and to refine species identification. With the plastid genome information, Qiu et al. reconstructed the phylogenetic relationship of the *Festuca*-*Lolium* complex. Synthetic or artificial polyploid hybrid materials within the *Triticum* genus or *Triticum* and its relative species represent essential wheat genetic improvement resources. Cytogenetic techniques can help provide insights into crop genomics, guiding further investigations on certain genomic issues. For example, to better characterize the tetraploid wheat-*Aegilops ventricosa* amphiploid materials, Zhang et al. observed the chromosomal behavior of the progeny plants (AABBD^V^D^V^N^V^N^V^) derived from crosses between *T. turgidum* (AABB) and *Aegilops ventricosa* (D^V^D^V^N^V^N^V^) using multicolor Fluorescence *in situ* hybridization (mc-FISH), providing insights into the genome stability of allopolyploidization in the wheat group.

## Genomic Technologies Enhance Crop Population Genetics

Genomic-based technologies have enhanced the traditional linkage mapping of quantitative trait loci (QTL) and enabled genome-wide association study (GWAS) by developing hundreds of thousands of markers (single nucleotide polymorphism, SNP, e.g., in most applications). In understudied crops, it is cost-effective to develop abundant SNP markers for QTL mapping by using reduced representation sequencing techniques, such as restriction-site associated DNA sequencing (RAD-seq) (Miller et al., 2007), genotyping-by-sequencing (GBS) (Elshire et al., 2011), and specific length amplified fragment sequencing (SLAF-seq) (Zhang et al., 2013). Wei et al. developed an interspecific F_2_ population containing 121 individuals, constructed a genetic map of eggplant (*Solanum melongena*) with 2,122 SNP markers and identified 19 QTLs for several morphological traits. This work lays a foundation for the fine mapping of QTLs and marker-assisted selection in eggplant breeding. In another work, Peng et al. used several SNP-identification methods (target enrichment sequencing, TES, RNA-seq and the 48K Axiom *Arachis*2 SNP array) to identify the genomic region and candidate genes controlling nodulation in cultivated peanut (*A. hypogaea* L.). They demonstrate that TES generated the highest number of SNPs, followed by RNA-seq and the SNP array with GBS being the least effective. In this work, TES and the SNP array have comparable costs per SNP per sample, while RNA-seq was the most expensive technique for SNP identification. To discover candidate genes associated with ear morphology in breeding populations, Li et al. identified SNPs for 208 maize inbred lines from two heterosis groups, Shaan A and Shaan B. The further GBS, combined GWAS and selective sweeps identified four genes associated with ear length and fruit length. Genomic technologies not only enhance QTL mapping, but also help in identifying expression QTL (eQTL). Barbey et al. identified SNPs for octoploid strawberry populations using the Affymetrix IStraw 35 Axiom SNP array and mapped 268 eQTLs for 224 genes expressed in the mature receptacle. Many of the eQTLs are known to affect fruit traits that were either described experimentally or validated via transgenic approaches.

## Genomic Technologies Enable Functional Mining of Genomic Components in Crops

Integration of multiple genomic resources, including but not limited to genetic variation by whole genome resequencing, gene expression by RNA-seq, miRNA expression by small RNA-seq and miRNA targets' cleavage information by degradome sequencing, can significantly enhance our understanding of transcriptional and post-transcriptional regulation in crops. Glazinska et al. created an expression database for yellow lupine (*Lupinus luteus* L.), namely LuLuDB, by combining RNA-seq analysis of small RNA, transcriptome, and degradome libraries, providing analysis-ready information of the NGS data. They further demonstrated the usefulness of the LuLuDB by a showcase of a genome-wide analysis of the *Dicer Like* (*DCL*) gene family and a *miR486*-*DCL2* analysis. In maize research, Xu et al. integrated 195 small RNA sequencing libraries and 19 degradome libraries. Together with the identification of phasi-RNA and GWAS results, they found many tissue-specific miRNAs and depicted evolutionary implications of small RNAs. Zhao et al. combined the heat-responsive transcriptomes of wheat and the genome-wide identified heat shock elements (HSEs) and show that a particular variant of non-canonical HSE is associated with a larger heat stress response and that the heat stress-responsive genes containing different HSEs are functionally diverged.

In addition to providing large-scale gene functional implications, genomic datasets can highlight a gene of interest within a particular gene family. For example, in a genome-wide analysis of wheat heat shock protein 90 (*TaHSP90*), Lu et al. took advantage of PacBio Iso-seq data and identified 126 isoforms derived from the *TaHSP90* genes. The highly expressed *TaHSP90-AA* genes showed a large magnitude of response to heat stress with differential alternative splicing patterns observed between the three *TaHSP90* homologous copies, extending our understanding of the functional divergence of the HSP family. Many pan-genome studies have revealed extensive genetic variations between accessions within a crop species, including copy number and structural variations. With the three high-quality phased diploid genomes of grapevine cultivars, Cabernet Sauvignon (CS), Carménère (CR), and Chardonnay (CH), Smit et al. compared the terpene synthase (*VviTPS*) gene family between CS, CR, CH, and an Illumina-based reference genome PN40024 (Jaillon et al., 2007; Chin et al., 2016; Minio et al., 2017, 2019; Roach et al., 2018). The in-depth genome-wide comparison of *VviTPS* family identified duplicated gene copies, predicted functions of *VviTPS* by combining sequence homology and established knowledge of more than 40 biochemically characterized *VviTPS* genes (Smit et al.) Zhang et al. summarized published genome-wide analyses of gene families in the cultivated and wild peanut (*Arachis*) genomes (Bertioli et al., 2016, 2019; Chen et al., 2016, 2019; Lu et al., 2018; Yin et al., 2018, 2019; Zhuang et al., 2019). Zhang et al. show that the hidden Markov Model (HMM)-based search of a gene family is rapid and accurate and provides helpful suggestions regarding aspects of gene family analysis.

The abundant genomic resources allow for investigation on the genomic components other than protein-coding genes and non-coding RNAs, such as untranslated regions (UTR) and *cis*-regulatory elements. Tu and Li (2020) developed an RNA-seq analysis method to profile alternative 3′ untranslated regions (3′UTRs), priUTR suitable for crops like sorghum and maize. Profiling of the genes with alternative 3′UTRs in *Sorghum bicolor* reveals a link between alternative 3′UTR and RNA N^6^-methyladenosine (m^6^A) modification, which had also been implicated in a previous maize RNA m^6^A profiling experiment (Luo et al., 2020). These papers provide bioinformatic evidence on the relationship between RNA m^6^A modification and alternative 3′UTRs/ polyadenylation. In 2021, a major breakthrough has been made to the link of m^6^A and alternative polyadenylation that the longer isoform of Cleavage and Polyadenylation Specificity Factor 30 (CPSF30-L) is the key protein to mediate m^6^A regulation of polyadenylation in *Arabidopsis* (Hou et al., 2021; Song et al., 2021). In this Research Topic, Galli et al. reviewed the state-of-art of our knowledge in regulatory regions and their mechanisms in controlling gene expression. Galli et al. further summarized the cutting-edge NGS technologies for detecting accessible chromatin regions (ACRs) and DNA-binding motifs of transcription factors (TFs). Particularly, the pros and cons of several methods for mapping TFs' DNA binding motifs [i.e., Chromatin immunoprecipitation sequencing (ChIP-seq), DNA Affinity Purification sequencing (DAP-seq) and cleavage under targets and release using nuclease (CUT&RUN)] have been discussed and highlighted. DAP-seq is considered a cost-efficient high-throughput method for crop regulome study. In addition, the reference genomes of closely related crops represent important resources for genome evolution studies. Yu et al. selected four pairs of genomes from the four core eudicot plant families, performed genome-wide synteny block comparison and discovered that excision of genes is much more prevalent than pseudogenization during genome fractionation.

## Concluding Remarks

The collection of sixteen papers in this Research Topic reflects the broad spectrum of current research directions in genomics-enabled crop genetics. The current Research Topic also exemplifies that genomic technologies and resources can be applied to a wide range of crop species, from cereal crops, such as wheat, maize and sorghum, to horticultural crops, such as eggplant, pistachio, yellow lupine, fine fescue, strawberry, and peanut. As the contributions to the Research Topic “Genomics-Enabled Crop Genetics” exemplarily shows, a combination of multiple genomic technologies and/or resources can form powerful and comprehensive tools for different aspects of crop genetic studies, suitable for different crop species with distinct applications and emphases. As more genomic resources and techniques are being developed for a variety of crop species, the output will accelerate crop genetic research and, ultimately, promote crop genetic improvement.

## Author Contributions

YL, CM, WW, and RM co-wrote this editorial based on this Research Topic's contributions. All authors contributed to the article and approved the submitted version.

## Conflict of Interest

The authors declare that the research was conducted in the absence of any commercial or financial relationships that could be construed as a potential conflict of interest.
